# Psychotropic Medication Exposure via Breast Milk: A Population‐Based Descriptive Study in Denmark

**DOI:** 10.1111/ppe.70074

**Published:** 2025-09-11

**Authors:** Xiaoqin Liu, Kathrine Bang Madsen, Jin Liang Zhu, Trine Munk‐Olsen, Per Damkier, Angela Lupattelli, Helga Zoega, Hedvig Nordeng, Mette‐Marie Zacher Kjeldsen, Merete Lund Mægbæk, Malene Galle Madsen, Veerle Bergink, Mette Bliddal

**Affiliations:** ^1^ NCRR‐The National Centre for Register‐Based Research, Department of Public Health Aarhus University Aarhus Denmark; ^2^ Research Unit of Child and Adolescent Psychiatry, Department of Clinical Research University of Southern Denmark Denmark; ^3^ Department of Clinical Pharmacology Odense University Hospital Odense Denmark; ^4^ Pharmacoepidemiology and Drug Safety Research Group, Department of Pharmacy University of Oslo Oslo Norway; ^5^ Centre of Public Health Sciences, Faculty of Medicine University of Oslo Iceland; ^6^ Department of Child Health and Development Norwegian Institute of Public Health Oslo Norway; ^7^ Department of Psychiatry Erasmus Medical Centre Rotterdam Rotterdam the Netherlands; ^8^ Department of Obstetrics, Gynecology and Reproductive Science Icahn School of Medicine at Mount Sinai New York USA; ^9^ Research Unit OPEN, Department of Clinical Research University of Southern Denmark Odense Denmark; ^10^ Clinical Pharmacology, Pharmacy and Environmental Medicine, Department of Public Health University of Southern Denmark Odense Denmark

**Keywords:** breastfeeding, cohort, epidemiology, psychotropic medication, register

## Abstract

**Background:**

There is limited data on the extent of psychotropic medication exposure through breast milk in infants. This information is essential for identifying research gaps and informing clinical practice.

**Objectives:**

To examine the prevalence and trend of psychotropic medication exposure among exclusively breastfed infants.

**Methods:**

A population‐based descriptive study among exclusively breastfed infants during 2012–2022, using Danish nationwide registers. Psychotropic prescriptions (Anatomical Therapeutic Chemical Classification System codes N05–N06) filled by mothers during the recorded breastfeeding period were identified in the Prescription Registry. We calculated the prevalence of potential exposure to any psychotropic medication (expressed per 1000 infants), categorised by drug class and stratified by maternal demographic and clinical factors.

**Results:**

Among 446,573 exclusively breastfed infants, 7882 (17.6 per 1000 infants, 95% confidence interval [CI] 17.2, 18.1) were exposed to at least one, and 699 (1.6 per 1000 infants, 95% CI 1.5, 1.7) to two different psychotropic medications via breastfeeding. The most frequent exposure was antidepressants, with a prevalence of 15.0 per 1000 infants (95% CI 14.6, 15.4), primarily sertraline. This was followed by hypnotics and sedatives, at 1.3 per 1000 infants (95% CI 1.2, 1.4), predominantly zopiclone, and antipsychotics, at 1.1 per 1000 infants (95% CI 1.0, 1.2), mainly quetiapine. Psychotropic medication exposure in exclusively breastfed infants increased 1.39‐fold, from 15.7 per 1000 infants (95% CI 14.5, 17.1) in 2012 to 21.8 per 1000 infants (95% CI 20.3, 23.4) in 2022. This increase was observed for all drug classes except anxiolytics. The prevalence of psychotropic medication exposure varied by maternal demographic and clinical factors.

**Conclusions:**

Approximately 2% of exclusively breastfed infants are potentially exposed to psychotropic medications through breast milk in Denmark. The prevalence has shown an upward trend over time, especially for psychostimulants.

## 1 Background

The beneficial effect of breastfeeding is well documented [1], including lower mortality and morbidity rates among breastfed infants and their mothers [2, 3]. The World Health Organization recommends exclusively breastfeeding infants for at least 6 months [4]. However, the decision to breastfeed can be complicated for mothers who are taking psychotropic medications. This is due to the potential risks of exposing the infants to these medications through breast milk, as well as the possibility of the mothers experiencing psychiatric relapses if medications are discontinued [5, 6, 7].

All psychotropic medications taken during lactation pass into breast milk to various degrees [8, 9, 10], and account for approximately 50% of reported adverse drug reactions among breastfed infants [11]. The amount of drug a nursing infant ingests varies substantially depending on the medication dose and pharmacokinetic properties [12, 13]. Currently, there are no regulatory guidelines specifying criteria for acceptable exposure in breastfed infants [14], and European product labels often advise against breastfeeding while using psychotropic medications due to the lack of data from human studies [15]. Only one Canadian cohort, the CHILD Study (2008–2012), comprising 3542 mother‐infant dyads, has reported prevalence estimates: at 3 months postpartum, 4.3% of breastfeeding women were using psychotropic medications [16].

Leveraging Danish national health registers, including detailed information on breastfeeding, we aimed to provide an overview of psychotropic medication exposure among exclusively breastfed infants and describe exposure by maternal demographic and health‐related characteristics.

## 2 Methods

### 2.1 Setting

We conducted a descriptive study on psychotropic medication exposure among infants in Denmark with records of exclusive breastfeeding, defined as receiving only breast milk, with the allowance of supplementation with water or similar liquids and/or a maximum of one meal with infant formula per week [17]. In Denmark, all residents are assigned a unique 10‐digit personal identifier (CPR number), which enables the linkage of individual‐level data between national registers [18]. The Danish National Child Health Register was established in 2009, and mandatory reporting to the registry was implemented in December 2011 [19]. The registry holds information on exclusive breastfeeding duration and anthropometric measurements of the children collected and recorded by general practitioners and healthcare nurses. The Danish Medical Birth Register holds information on all births in Denmark and enables the identification of all liveborn children linked to their biological mothers [20, 21]. Since 1995, the Danish National Prescription Registry has included individual‐level information on all filled prescriptions at community pharmacies in Denmark [22]. The Danish National Patient Registry [23] contains data on all in‐ and outpatient contacts since 1995, including the International Classification of Diseases, version 10 (ICD‐10) diagnoses. The Danish Psychiatric Central Research Register [24] was established as an electronic database in 1969. The register holds information on all treatments at psychiatric hospitals and psychiatric wards in general hospitals since 1969. In 1995, data on emergency room visits and outpatient treatments were included. Importantly, diagnoses made by general practitioners are not recorded in either the Danish National Patient Registry or the Danish Psychiatric Central Research Register.

### 2.2 Exclusive Breastfeeding

We obtained individual‐level information on exclusive breastfeeding for each child registered in the Danish National Child Health Register. Denmark has a tax‐funded healthcare system with free access to healthcare, and healthcare nurses offer at least five free home visits to all newborns during the first year of life. The healthcare nurses report information prospectively on the duration and end date of exclusive breastfeeding at routine home visits in the first year after childbirth, with data coverage becoming complete and more reliable from 2012 [19]. The increase observed in 2012–2014, as shown in Figure S1, likely corresponds to this transition period, during which the number of municipalities contributing high‐quality data increased substantially, rather than indicating a sudden population‐level shift in breastfeeding practices. Exclusive breastfeeding is recorded if the infant receives only breast milk in addition to water and no more than one bottle of infant formula per week after hospital discharge [17]. The exclusive breastfeeding period (in days) was defined from the date of birth until the end date of exclusive breastfeeding recorded by the health nurses based on maternal reporting. Notably, infants who were partially breastfed were not recorded as exclusively breastfed in the register and, therefore, were not included in the study.

### 2.3 Study Population

**FIGURE 1:**
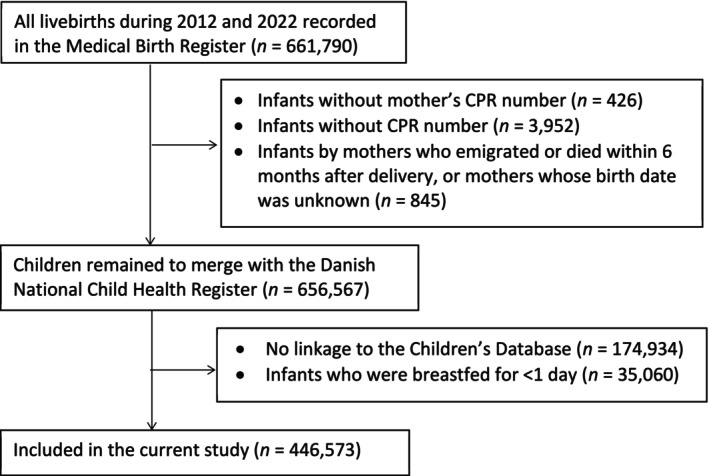
Flowchart of the study population.

We identified 661,790 liveborn infants and their mothers in the Danish Medical Birth Register from January 1, 2012, to December 31, 2022 (see Figure 1, Flowchart). We excluded 426 infants with unknown maternal CPR numbers, 3952 infants with unknown CPR numbers, and 845 infants whose mothers emigrated or died within 6 months after delivery or whose mothers' birth dates were unknown. Using the infant's identification number, we linked each child to the Danish National Child Health Register, which contains data only on exclusively breastfed infants. We then further excluded 209,994 infants: 174,934 were not linked to the Danish National Child Health Register, and 35,060 were breastfed for less than 1 day, indicating no breastfeeding initiation. A total of 446,573 infants (including 15,888 multiple births) born by 329,276 mothers remained in the analysis. The characteristics of the included infants and 209,994 excluded infants from the analysis are largely comparable, as detailed in Table S1.

### 2.4 Psychotropic Medication Exposure

Psychotropic exposure among exclusively breastfed infants was defined as any maternal prescriptions of a psychotropic medication filled between the child's birth and the last date of exclusive breastfeeding, thereby capturing maternal prescriptions that overlapped with the breastfeeding period. Prescriptions filled less than 7 days before the end of breastfeeding were excluded to minimise misclassification of exposure among infants of mothers starting on psychotropic medication after the end of breastfeeding. Information on filled psychotropic prescriptions was extracted from the Danish Prescription Registry using Anatomical Therapeutic Chemical Classification (ATC) codes N05 and N06. The following ATC codes were used to define drug classes: antipsychotics (N05A), anxiolytics (N05B), hypnotics and sedatives (N05C), antidepressants (N06A), and psychostimulants (N06B). Detailed ATC codes for drug classes and the most common individual drugs can be found in Table S2.

### 2.5 Statistical Analysis

The prevalence of psychotropic medication exposure was estimated for any psychotropic medication and by ATC drug class. We first calculated the prevalence by dividing the number of infants potentially exposed to psychotropic medications during exclusive breastfeeding by the total number of exclusively breastfed infants expressed per 1000 infants. This was done overall and per calendar year of birth. As per the definition, infants whose mothers filled more than one psychotropic drug class prescription were only counted once in the calculation of the prevalence of any psychotropic prescription exposure, but contributed to each drug class if potentially exposed. We identified the five most common psychotropic medication exposures within each drug class by ATC level 5. We estimated 95% confidence intervals (CIs) by performing cluster bootstrapping with 1000 resamples, resampling at the maternal level to properly account for dependencies among siblings.

To explore whether the prevalence varied by maternal characteristics, we stratified analyses by maternal age at childbirth, primiparity, education level, cohabitant status, country of origin, birth outcomes (caesarean section, low birthweight, preterm birth, and multiple/singleton birth), and calendar year of delivery. Information on these characteristics was derived from the Danish Medical Birth Registry and Statistics Denmark. The detailed definition of birth outcomes can be found in the eMethods in the Supporting Information. The prevalence of psychotropic medication exposure was stratified by maternal clinical features, including psychiatric diagnosis and prior psychotropic medication use (≥ 1 prescription fill) within the last 5 years before the index delivery. Maternal psychiatric diagnosis was extracted from the Danish Psychiatry Central Research Register and the Danish National Patient Registry using ICD‐10 codes F00 to F99 (Table S3).

To assess how the prevalence varied with different definitions of psychotropic medication exposure via breastfeeding, we repeated the analyses using (1) a more inclusive definition of maternal filling of a psychotropic prescription from 7 days before childbirth through the last day of exclusive breastfeeding, without excluding prescriptions filled within 7 days before the end of breastfeeding and (2) maternal filling of at least one prescription for any psychotropic medication from the date of birth to 14 days before the last date of exclusive breastfeeding. All analyses were performed in Stata 15.0.

### 2.6 Missing Data

Approximately 3.4% of exclusively breastfed infants had missing data on one or more covariates related to maternal characteristics or clinical features. Given the descriptive nature of the study, we retained these observations by applying a missing indicator method.

## 3 Results

**FIGURE 2:**
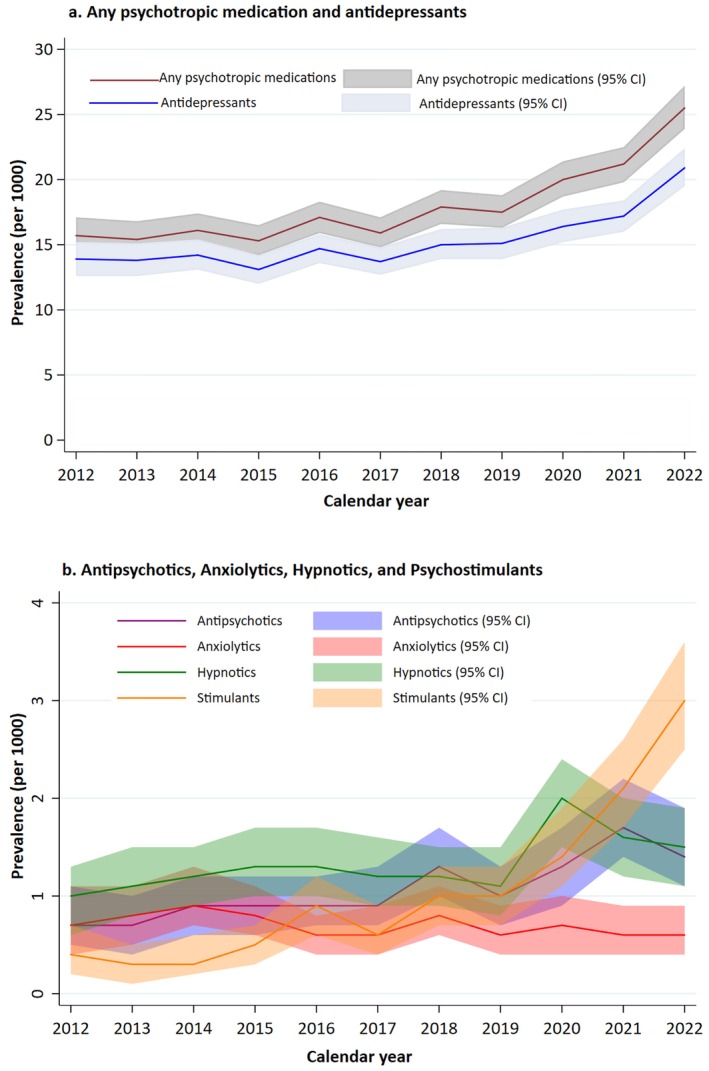
Prevalence of psychotropic drug exposure among 446,573 exclusively breastfed infants (overall and by medication classes) over the calendar year. To be noted: The value of the y‐axis differs between Panels a and b.

The study included 446,573 exclusively breastfed infants born by 329,276 mothers. The mean age (SD) of the mothers was 30.9 (4.9) years, and 46.9% were primiparous. The median duration of breastfeeding was 130 days (interquartile range: 48 to 167), with 34% of infants exclusively breastfed for fewer than 90 days. The prevalence of exclusive breastfeeding by calendar year is shown in Figure S1. Of the exclusively breastfed infants, 7882 (17.6 per 1000 infants, 95% CI: 17.2, 18.1) were potentially exposed to any psychotropic medication via breast milk, and 699 (1.6 per 1000 infants, 95% CI: 1.5, 1.7) to ≥ 2 different psychotropic medications. The prevalence of exposure increased by 1.39‐fold from 15.7 (2012) to 21.8 per 1000 infants (2022) (Figure 2 and Table 1).

**TABLE 1:** Prevalence (95% CI) of psychotropic medication exposure among 446,573 exclusively breastfed infants in Denmark during 2012–2022.

Year	Number of exclusively breastfed children	Potentially exposed to any psychotropic medication		Potentially exposed to two or more medications	
		*N*	Prevalence and 95% CIs (per 1000 infants)	*N*	Prevalence and 95% CIs (per 1000 infants)
2012–2022	446,573	7882	17.6 (17.2, 18.1)	699	1.6 (1.5, 1.7)
2012	32,616	513	15.7 (14.5, 17.1)	46	1.4 (1.1, 1.9)
2013	34,656	535	15.4 (14.2, 16.8)	50	1.4 (1.1, 1.9)
2014	40,452	653	16.1 (15.0, 17.4)	53	1.3 (1.0, 1.7)
2015	43,208	661	15.3 (14.2, 16.5)	61	1.4 (1.1, 1.8)
2016	44,731	764	17.1 (15.9, 18.3)	72	1.6 (1.3, 2.0)
2017	44,726	712	15.9 (14.9, 17.0)	56	1.3 (1.0, 1.6)
2018	42,637	762	17.9 (16.7, 19.2)	63	1.5 (1.2, 1.9)
2019	42,580	746	17.5 (16.3, 18.8)	63	1.5 (1.2, 1.9)
2020	40,380	809	20.0 (18.7, 21.5)	79	2.0 (1.6, 2.4)
2021	44,064	932	21.2 (19.8, 22.6)	92	2.1 (1.7, 2.6)
2022	36,523	795	21.8 (20.3, 23.4)	64	1.8 (1.4, 2.2)

The most common psychotropic class exposure among exclusively breastfed infants was antidepressants (15.0 per 1000 infants), followed by hypnotics and sedatives (1.3 per 1000 infants), antipsychotics (1.1 per 1000 infants), psychostimulants (1.0 per 1000 infants), and anxiolytics (0.7 per 1000 infants). When comparing the prevalence of exposure in 2012 vs. 2022, we observed an increase for antidepressants (1.28‐fold from 13.9 to 17.7 per 1000 infants), antipsychotics (1.71‐fold from 0.7 to 1.3 per 1000 infants), psychostimulants (6.80‐fold from 0.4 to 2.7 per 1000 infants), and hypnotics and sedatives (1.27‐fold from 1.0 to 1.2 per 1000 infants), but not for anxiolytics (0.74‐fold from 0.7 to 0.5 per 1000 infants).

Table 2 presents the prevalence of the five most frequent psychotropic medication exposures by drug class. For antidepressants, sertraline was the most common exposure via breastfeeding among infants (56.0%), followed by citalopram (17.7%). Among psychostimulants, methylphenidate accounted for most exposures (89.0%). For antipsychotics, quetiapine was the most frequent exposure (63.3%), followed by olanzapine (15.4%). Within hypnotics and sedatives, zopiclone accounted for about half of the prescriptions (52.5%), and within anxiolytics, oxazepam was the medication to which most infants were potentially exposed via breast milk (72.4%).

**TABLE 2:** Most commonly exposed psychotropic medications within each drug class via breastfeeding among 446,573 exclusively breastfed infants.

Drug classes	*N*	Prevalence and 95% CIs (per 1000 infants)	% of exposure within drug classes
Antipsychotics	472	1.1 (1.0, 1.2)	100.0
Quetiapine	309	0.7 (0.6, 0.8)	63.3
Olanzapine	75	0.2 (0.1, 0.2)	15.4
Chlorprothixene	26	0.1 (0.0, 0.1)	5.3
Risperidone	26	0.1 (0.0, 0.1)	5.3
Lithium	16	0.0 (0.0, 0.1)	3.3
Anxiolytics	309	0.7 (0.6, 0.8)	100.0
Oxazepam	228	0.5 (0.4, 0.6)	72.4
Diazepam	34	0.1 (0.1, 0.1)	10.8
Alprazolam	32	0.1 (0.1, 0.1)	10.2
Clobazam	13	0.0 (0.0, 0.1)	4.1
Hypnotics and sedatives	577	1.3 (1.2, 1.4)	100.0
Zopiclone	317	0.7 (0.6, 0.8)	52.5
Zolpidem	158	0.4 (0.3, 0.4)	26.2
Melatonin	111	0.2 (0.2, 0.3)	18.4
Triazolam	12	0.0 (0.0, 0.1)	2.0
Antidepressants	6685	15.0 (14.6, 15.4)	100.0
Sertraline	3863	8.7 (8.4, 9.0)	56.0
Citalopram	1223	2.7 (2.6, 2.9)	17.7
Venlafaxine	701	1.6 (1.4, 1.7)	10.2
Escitalopram	229	0.5 (0.4, 0.6)	3.3
Nortriptyline	196	0.4 (0.4, 0.5)	2.8
Psychostimulants	457	1.0 (0.9, 1.1)	100.0
Methylphenidate	412	0.9 (0.8, 1.0)	89.0
Lisdexamfetamine	29	0.1 (0.0, 0.1)	6.3
Atomoxetine	16	0.0 (0.0, 0.1)	3.5

*Note:* The percentages do not add up to 100% because a single child can be exposed to multiple psychotropic medications through breastfeeding.

Table 3 and Table S4 show the prevalence and 95% CIs of any psychotropic medication exposure overall and by medication classes according to maternal characteristics and clinical features. The prevalence of exposure to any psychotropic medication via breastfeeding increased with maternal age at childbirth and duration of breastfeeding. It was higher among infants of Danish‐born mothers compared to those of non‐Danish‐born mothers, and among infants born with adverse birth outcomes compared to those without. The most significant difference was observed in mothers with a history of psychiatric disorders treated in hospitals before delivery. The prevalence was 110.1 per 1000 infants (95% CI: 106.4, 113.9) among mothers with a diagnosed psychiatric disorder within the previous 5 years, compared to 11.2 per 1000 infants (95% CI: 10.8, 11.5) among those without. A similar pattern was seen for prior psychotropic medication use, with a prevalence of 106.5 per 1000 infants (95% CI: 103.9, 109.1) among those with prior use, compared to 2.3 per 1000 infants (95% CI: 2.1, 2.4) among those without. Similar associations were observed within all drug classes, except that the highest prevalence of psychostimulant exposure was observed in mothers aged < 25 years and decreased with increasing maternal age.

**TABLE 3:** Prevalence (95% CIs) of potential psychotropic medication exposure among 446,573 exclusively breastfed infants by maternal characteristics and clinical features in Denmark, 2012–2022.

Characteristics	No. of children	Any psychotropic medications (per 1000 infants)	Antipsychotics (per 1000 infants)	Anxiolytics (per 1000 infants)	Hypnotics and sedatives (per 1000 infants)	Antidepressants (per 1000 infants)	Psychostimulants (per 1000 infants)
Duration of breastfeeding (months)							
< 1	87,745	3.0 (2.6, 3.3)	0.3 (0.2, 0.5)	0.2 (0.1, 0.3)	0.4 (0.3, 0.5)	2.1 (1.9, 2.5)	0.3 (0.2, 0.4)
1–3	88,129	17.8 (16.9, 18.7)	1.2 (1.0, 1.4)	0.7 (0.6, 0.9)	1.6 (1.4, 1.9)	14.3 (13.5, 15.1)	1.5 (1.3, 1.8)
4–5	181,778	22.0 (21.3, 22.7)	1.2 (1.1, 1.4)	0.8 (0.7, 1.0)	1.4 (1.3, 1.6)	19.1 (18.4, 19.8)	1.1 (1.0, 1.3)
≥ 6	88,921	23.1 (22.1, 24.1)	1.3 (1.1, 1.6)	1.0 (0.8, 1.2)	1.6 (1.3, 1.9)	19.8 (18.9, 20.8)	1.1 (0.9, 1.3)
Maternal age at childbirth (years)							
< 25	48,013	13.0 (12.0, 14.1)	1.1 (0.9, 1.5)	0.5 (0.3, 0.7)	0.6 (0.4, 0.9)	10.1 (9.2, 11.1)	1.9 (1.5, 2.3)
25–29	150,409	14.9 (14.3, 15.6)	0.8 (0.7, 1.0)	0.5 (0.4, 0.7)	1.0 (0.8, 1.1)	12.5 (11.9, 13.2)	1.1 (1.0, 1.3)
30–34	157,705	18.7 (18.0, 19.4)	1.1 (0.9, 1.3)	0.7 (0.6, 0.9)	1.5 (1.3, 1.7)	16.0 (15.4, 16.7)	0.9 (0.7, 1.0)
≥ 35	90,446	22.8 (21.8, 23.8)	1.3 (1.1, 1.6)	1.0 (0.8, 1.2)	1.9 (1.6, 2.2)	19.8 (18.9, 20.8)	0.7 (0.5, 0.9)
Primiparous							
Yes	209,440	17.5 (16.9, 18.1)	1.1 (0.9, 1.2)	0.7 (0.6, 0.8)	1.5 (1.3, 1.7)	14.4 (13.9, 14.9)	1.2 (1.1, 1.4)
No	237,133	17.8 (17.2, 18.4)	1.1 (0.9, 1.2)	0.7 (0.6, 0.8)	1.1 (1.0, 1.3)	15.5 (15.0, 16.0)	0.8 (0.7, 1.0)
Pregnancy							
Singletons	430,685	17.8 (17.4, 18.3)	1.1 (1.0, 1.2)	0.7 (0.6, 0.8)	1.3 (1.2, 1.4)	15.1 (14.7, 15.5)	1.0 (0.9, 1.2)
Multiple births	15,888	13.0 (10.9, 15.4)	0.5 (0.2, 1.1)	0.8 (0.4, 1.4)	1.5 (0.9, 2.5)	10.5 (8.6, 12.8)	0.4 (0.1, 1.0)
Maternal country of origin							
Danish	359,407	19.0 (18.5, 19.5)	1.1 (0.9, 1.2)	0.7 (0.6, 0.8)	1.3 (1.2, 1.4)	16.2 (15.7, 16.6)	1.2 (1.1, 1.3)
Non‐Danish, including missing	87,166	12.0 (11.2, 12.8)	1.1 (0.9, 1.4)	0.6 (0.5, 0.8)	1.2 (1.0, 1.5)	10.0 (9.3, 10.7)	0.4 (0.3, 0.6)
Maternal smoking during pregnancy							
No	392,660	16.8 (16.3, 17.2)	1.0 (0.9, 1.1)	0.7 (0.6, 0.8)	1.3 (1.2, 1.4)	14.4 (14.0, 14.8)	0.8 (0.7, 0.9)
Yes	40,000	26.1 (24.4, 27.8)	2.0 (1.6, 2.4)	0.9 (0.6, 1.3)	1.0 (0.7, 1.3)	20.9 (19.4, 22.4)	3.1 (2.5, 3.7)
Missing	13,913	18.0 (15.8, 20.5)	1.0 (0.6, 1.7)	0.6 (0.3, 1.2)	1.4 (0.9, 2.1)	15.4 (13.4, 17.6)	1.1 (0.7, 1.7)
Maternal cohabitant status in the year of delivery							
Married or cohabiting	411,991	17.2 (16.7, 17.6)	1.0 (0.9, 1.1)	0.7 (0.6, 0.8)	1.3 (1.2, 1.4)	14.7 (14.3, 15.1)	0.9 (0.8, 1.0)
Single, divorced, or widowed^a^	34,582	23.4 (21.8, 25.1)	2.0 (1.6, 2.5)	0.9 (0.7, 1.3)	1.3 (1.0, 1.8)	18.0 (16.5, 19.6)	2.8 (2.3, 3.5)
Maternal education							
Mandatory school, including missing	69,692	19.9 (18.8, 21.0)	1.9 (1.6, 2.2)	0.7 (0.5, 0.9)	0.9 (0.7, 1.2)	15.5 (14.5, 16.5)	2.4 (2.1, 2.9)
High school or vocational school	159,422	17.5 (16.9, 18.2)	1.0 (0.9, 1.2)	0.7 (0.6, 0.8)	0.9 (0.7, 1.1)	15.1 (14.4, 15.7)	1.1 (1.0, 1.3)
College or university	217,459	17.0 (16.4, 17.6)	0.8 (0.7, 1.0)	0.7 (0.6, 0.8)	1.7 (1.5, 1.9)	14.7 (14.2, 15.3)	0.5 (0.4, 0.6)
Caesarean section							
No	358,601	17.1 (16.6, 17.6)	1.0 (0.9, 1.1)	0.6 (0.6, 0.7)	1.3 (1.2, 1.4)	14.5 (14.1, 15.0)	1.0 (0.9, 1.1)
Yes	87,972	20.0 (19.0, 21.0)	1.3 (1.1, 1.6)	0.9 (0.7, 1.1)	1.4 (1.1, 1.6)	16.8 (15.9, 17.8)	1.1 (0.9, 1.4)
Preterm birth							
No	419,342	17.0 (16.5, 17.4)	N/A	N/A	N/A	14.3 (13.9, 14.7)	N/A
Yes	23,504	31.0 (28.7, 33.4)	N/A	N/A	N/A	27.6 (25.4, 29.9)	N/A
Missing	3727	9.9 (7.1, 13.9)	N/A	N/A	N/A	8.0 (5.5, 11.8)	N/A
Low birthweight							
No	423,888	17.4 (16.9, 17.8)	N/A	N/A	1.3 (1.2, 1.4)	14.7 (14.3, 15.1)	N/A
Yes	17,863	26.9 (24.5, 29.5)	N/A	N/A	1.8 (1.2, 2.6)	22.8 (20.5, 25.4)	N/A
Missing	4822	9.3 (6.9, 12.7)	N/A	N/A	1.0 (0.4, 2.4)	7.5 (5.3, 10.6)	N/A
Maternal psychiatric diagnosis within 5 years before childbirth							
No	417,268	11.2 (10.8, 11.5)	0.4 (0.3, 0.4)	0.5 (0.5, 0.6)	1.1 (1.0, 1.2)	9.4 (9.1, 9.7)	0.6 (0.5, 0.6)
Yes	29,305	110.1 (106.4, 113.9)	10.9 (9.7, 12.3)	2.9 (2.3, 3.5)	3.8 (3.2, 4.6)	94.3 (90.7, 98.1)	7.7 (6.8, 8.8)
Substance abuse disorder							
No	444,066	17.5 (17.1, 18.0)	1.0 (0.9, 1.1)	N/A	N/A	14.9 (14.5, 15.3)	1.0 (0.9, 1.1)
Yes	2507	37.1 (30.3, 45.4)	6.4 (4.0, 10.2)	N/A	N/A	27.5 (21.5, 35.1)	4.0 (2.2, 7.3)
Schizophrenia disorders							
No	445,063	17.3 (16.9, 17.8)	0.9 (0.8, 1.0)	0.7 (0.6, 0.8)	1.3 (1.2, 1.4)	14.8 (14.4, 15.2)	1.0 (0.9, 1.1)
Yes	1510	113.2 (97.5, 131.1)	55.0 (44.2, 68.2)	4.0 (1.8, 8.8)	7.9 (4.4, 14.4)	58.9 (48.1, 72.1)	6.6 (3.3, 13.2)
Mood disorders							
No	439,038	14.6 (14.2, 15.0)	0.7 (0.6, 0.8)	0.6 (0.6, 0.7)	1.2 (1.1, 1.3)	12.2 (11.9, 12.5)	1.0 (0.9, 1.1)
Yes	7535	194.6 (185.2, 204.3)	20.3 (17.2, 23.9)	4.5 (3.2, 6.3)	6.0 (4.4, 8.1)	176.5 (167.3, 186.1)	4.5 (3.2, 6.3)
Anxiety and stress‐related disorders							
No	431,983	14.7 (14.3, 15.1)	0.8 (0.7, 0.9)	0.6 (0.5, 0.7)	1.2 (1.1, 1.3)	12.3 (11.9, 12.6)	0.9 (0.8, 1.0)
Yes	14,590	106.4 (101.2, 111.9)	8.4 (7.0, 10.0)	3.8 (2.9, 4.9)	4.0 (3.1, 5.2)	95.0 (90.0, 100.2)	3.9 (3.0, 5.1)
Personality disorders							
No	441,590	16.7 (16.3, 17.2)	0.9 (0.8, 1.0)	0.7 (0.6, 0.8)	1.3 (1.2, 1.4)	14.2 (13.8, 14.6)	1.0 (0.9, 1.1)
Yes	4983	97.9 (89.6, 106.9)	13.4 (10.4, 17.4)	1.6 (0.8, 3.2)	2.2 (1.2, 3.9)	82.5 (74.7, 91.0)	6.6 (4.7, 9.4)
Autism spectrum disorders							
No	446,364	17.6 (17.2, 18.0)	N/A	N/A	N/A	14.9 (14.5, 15.4)	N/A
Yes	209	114.8 (76.9, 168.1)	N/A	N/A	N/A	95.7 (61.8, 145.2)	N/A
Behavioural and emotional disorders with onset usually occurring in childhood and adolescence							
No	444,283	17.2 (16.7, 17.6)	1.0 (0.9, 1.1)	N/A	1.3 (1.2, 1.4)	14.8 (14.4, 15.2)	0.7 (0.6, 0.8)
Yes	2290	111.8 (99.1, 125.9)	8.7 (5.7, 13.4)	N/A	3.5 (1.6, 7.6)	43.7 (36.2, 52.6)	69.9 (59.5, 81.9)
Other psychiatric disorders							
No	441,951	17.0 (16.6, 17.4)	1.0 (0.9, 1.1)	0.7 (0.6, 0.8)	1.2 (1.1, 1.4)	14.4 (14.1, 14.8)	1.0 (0.9, 1.1)
Yes	4622	79.2 (71.1, 88.1)	9.3 (6.9, 12.6)	3.0 (1.8, 5.2)	5.4 (3.6, 8.2)	66.2 (59.0, 74.2)	5.6 (3.7, 8.6)
Any psychotropic medication within 5 years before childbirth							
No	380,649	2.3 (2.1, 2.4)	0.2 (0.2, 0.2)	0.3 (0.3, 0.4)	0.8 (0.7, 0.9)	1.4 (1.3, 1.5)	0.0 (0.0, 0.1)
Yes	65,924	106.5 (103.9, 109.1)	6.0 (5.4, 6.7)	2.9 (2.5, 3.3)	4.4 (3.8, 5.0)	93.4 (91.0, 96.0)	6.8 (6.1, 7.4)
Antipsychotics within 5 years before childbirth							
No	436,771	14.8 (14.4, 15.2)	0.3 (0.3, 0.4)	0.6 (0.5, 0.7)	1.2 (1.1, 1.3)	12.8 (12.4, 13.2)	0.8 (0.7, 0.9)
Yes	9802	145.8 (138.3, 153.6)	34.2 (30.7, 38.1)	4.4 (3.2, 6.0)	7.0 (5.5, 9.0)	112.4 (105.7, 119.5)	9.6 (7.8, 11.8)
Anxiolytics within 5 years before childbirth							
No	435,114	15.3 (14.9, 15.7)	0.8 (0.8, 0.9)	0.4 (0.4, 0.5)	1.2 (1.1, 1.3)	13.0 (12.6, 13.3)	0.9 (0.8, 1.0)
Yes	11,459	108.4 (102.3, 114.8)	9.2 (7.4, 11.3)	10.2 (8.4, 12.3)	5.5 (4.2, 7.2)	91.1 (85.6, 96.9)	5.1 (3.9, 6.6)
Hypnotics and sedatives within 5 years before childbirth							
No	427,168	14.6 (14.2, 15.0)	0.7 (0.7, 0.8)	0.6 (0.5, 0.6)	0.9 (0.8, 1.0)	12.6 (12.3, 13.0)	0.7 (0.6, 0.8)
Yes	19,405	84.4 (80.3, 88.7)	7.9 (6.7, 9.4)	3.3 (2.6, 4.2)	9.7 (8.3, 11.3)	66.2 (62.6, 70.0)	7.7 (6.5, 9.2)
Antidepressants within 5 years before childbirth							
No	400,832	3.5 (3.3, 3.7)	0.5 (0.4, 0.6)	0.4 (0.4, 0.5)	1.0 (0.9, 1.1)	1.5 (1.4, 1.6)	0.7 (0.6, 0.7)
Yes	45,741	141.4 (137.9, 145.0)	6.1 (5.4, 6.8)	3.0 (2.6, 3.6)	4.0 (3.4, 4.6)	132.9 (129.5, 136.4)	4.2 (3.7, 4.9)
Psychostimulants within 5 years before childbirth							
No	441,278	16.2 (15.8, 16.7)	1.0 (0.9, 1.1)	0.7 (0.6, 0.8)	1.3 (1.2, 1.4)	14.4 (14.0, 14.9)	0.0 (0.0, 0.1)
Yes	5295	136.0 (126.4, 146.2)	5.7 (3.9, 8.1)	1.7 (0.9, 3.3)	3.8 (2.4, 6.0)	58.5 (52.0, 65.9)	82.9 (75.5, 90.9)

*Note:* N/A: not applicable due to fewer than 5 cases, or not relevant.

^a^ Fewer than five individuals have missing data on cohabitant status in the drug class analysis, and we merged them into the single, divorced, or widowed group.

In two sensitivity analyses employing alternative definitions of psychotropic medication exposure, we observed similar trends by calendar year consistent with the main analysis. First, when defining psychotropic medication exposure as prescription fills from the 7 days prior to childbirth until the end of exclusive breastfeeding, the prevalence of exposure was only slightly higher for overall drug exposure at 20.2 per 1000 infants compared to 17.6 per 1000 infants found in the main analysis (Table S5). Second, when psychotropic medication exposure was defined as at least one maternal prescription fill from the infant's birth until 14 days before the end of exclusive breastfeeding, the prevalence of exposure was slightly lower, recorded at 15.9 per 1000 infants (Table S6).

## 4 Comment

### 4.1 Principal Findings

In this population‐based descriptive study of 446,573 exclusively breastfed infants, one in 57 infants was potentially exposed to a psychotropic medication via breast milk. The prevalence increased 1.39‐fold during 2012–2022 (from 15.7 to 21.8 per 1000 infants), driven mainly by antidepressants. This upward trend was observed across antipsychotics, hypnotics and sedatives, antidepressants, and psychostimulants, while the prevalence of anxiolytics remained stable over time. The observed patterns described here suggest that medications were largely prescribed following changes in clinical guidelines and recommendations [14]. Prevalence varied by maternal characteristics. The largest difference in exposure prevalence was observed among infants whose mothers had a prior psychiatric diagnosis or a history of psychotropic medication treatment.

### 4.2 Strengths of the Study

This study comprises a large representative population. The linkage between the Danish National Child Health Register and the nationwide registers on birth records, filled prescriptions, hospital treatment, and socioeconomic status gives an exceptional opportunity to provide a detailed description of psychotropic prescription exposure among exclusively breastfed infants. Our findings provide a comprehensive overview and lay the foundation for future research. The registers used to retrieve data on maternal characteristics, clinical factors, and filled prescriptions are extensive, almost complete, and well‐validated.

### 4.3 Limitations of the Data

Our study also has some limitations. First, we included only exclusively breastfed infants, who accounted for approximately 68% of all infants born during the study period. It is estimated that over 80% of new mothers in Denmark breastfeed their infants in the first days of life [25], which indicates that not all exclusively breastfed infants are included in our study, potentially due to a lack of reporting in the Danish National Child Health Register. However, as indicated by our findings of minimal differences between included and non‐included infants on maternal characteristics and clinical factors, this should not affect prevalence estimates. Additionally, an increase in exclusive breastfeeding rates between 2012 and 2014 was observed, most likely attributable to improved data coverage across municipalities during this period. Since this variation is unrelated to psychotropic medication prescription, it is unlikely to have influenced the prevalence estimates. Second, the breastfeeding data in the Danish Child Health Register have not been validated in large‐scale validation studies, which may introduce some uncertainty regarding data accuracy and completeness. Third, our study measures medication prescriptions during breastfeeding. Women who fill a prescription do not necessarily take the medications, and we may, therefore, have overestimated the prevalence of exposed infants who were exclusively breastfed. Conversely, the use of psychotropics while partially breastfeeding is not documented in the Danish National Child Health Register, which might underestimate the proportion of infants exposed to psychotropics while breastfeeding. Lastly, Denmark has a tax‐funded healthcare system with free access to healthcare. Our findings may not be generalisable to other populations with different healthcare systems and guidelines for the duration of breastfeeding in other countries.

### 4.4 Interpretation

Our study investigated psychotropic medication exposure among exclusively breastfed infants in a large and representative population. Previous studies have primarily involved small samples [10]. Our findings of increased psychotropic medication exposure align with previous findings among women of reproductive age [26, 27, 28]. The observed upward trend could be attributed to the general increase in the prevalence of psychiatric disorders [29], more psychotropic medication use in women of reproductive age, and the rising number of women with mental disorders giving birth [30]. During the study period, recommendations and guidelines have changed toward a less restrictive approach for the use of psychotropics during lactation as safety data continuously improve [8, 10, 12, 14, 31, 32]. Consequently, mothers may perceive these medications as safe to use while breastfeeding. Lastly, we observed a marked increase since 2020, coinciding with the onset of the COVID‐19 pandemic, a period that likely affected both maternal mental health and healthcare utilisation patterns [33], and consequently, the prevalence of potential psychotropic medication exposure.

Databases such as LactMed [34] and Janusinfo [35], and National Information Services play a key role in providing healthcare professionals with evidence‐based information about medications and breastfeeding. Most consider *relative infant dose* (RID), which quantitatively assesses drug concentration in breast milk as related to maternal dose. While there is no regulatory or formally agreed‐upon academic or clinical threshold, an RID of less than 10% is generally considered to be compatible with breastfeeding unless the medication has high toxicity. In Denmark, the most important decision support system (www.pro.medicin.dk; an analogue to the Physicians' Desk Reference) has adopted a RID of 5% as a principal cut‐off.

We found that antidepressants were the most used psychotropic medication by mothers of potentially exposed infants, increasing 1.28‐fold from 2012 to 2022. Sertraline was the most commonly used antidepressant, followed by citalopram, venlafaxine, escitalopram, and nortriptyline, in line with our previous study among pregnant women [36]. Most antidepressants, such as sertraline, escitalopram, and nortriptyline, do not expose nursing infants to clinically important amounts through breastfeeding (RID < 10%), and small studies suggest no short‐term adverse effects associated with these antidepressants during breastfeeding [10]. Other antidepressants, such as fluoxetine [37], citalopram [38], and venlafaxine [39], may lead to higher systemic infant exposure (RID reaching 10%). Our findings show that these drugs with high RID were also prescribed during breastfeeding. For instance, 1.6 out of 1000 infants were potentially exposed to venlafaxine via breastfeeding. While there is no strong evidence suggesting potential adverse effects on nursing infants [10, 40], it is important to note that the long‐term outcomes of antidepressant exposure through breastfeeding have not been thoroughly studied. However, a recent expert consensus statement concludes that pharmacological treatment for depressive and anxiety disorders, such as selective serotonin reuptake inhibitors (SSRIs), is not contraindicated for breastfeeding [41].

The increase in antipsychotic exposure aligns with trends observed in women of childbearing age and among pregnant women [26, 42]. Many antipsychotics have a very low RID (less than 1%), except for haloperidol and risperidone [8], which are used very limitedly during breastfeeding, according to our findings. Only a few prospective studies assessed the safety of antipsychotics during breastfeeding, with a limited number of exposed infants [8].

Psychostimulants are effective in reducing core symptoms of attention‐deficit/hyperactivity disorder (ADHD) in the short term [43] and have also been shown to reduce the long‐term risk of substance use disorders and accidents among individuals with ADHD [44, 45]. Psychostimulants are increasingly prescribed to women of reproductive age [27, 28]. Our findings of increased psychostimulants use during breastfeeding align with this overall trend, which may reflect an increasing awareness of ADHD in women among clinicians and recognition of the disorder's persistence into adulthood [46, 47]. From 2012 to 2022, psychostimulant medication use among women of reproductive age increased 3‐fold [48], whereas we observed a 6.80‐fold increase among exclusively breastfed infants. Evidence, primarily from case studies, indicates that when prescribed for medical indications, methylphenidate levels in breast milk are very low and typically undetectable in the serum of infants [49, 50, 51]. These studies also suggested that infants exposed to methylphenidate showed no adverse reactions related to the drug and were developing normally for their age in the short term [49, 50, 51]. For amphetamines, less data is available, and most clinical guidelines recommend caution [52]. Moreover, the long‐term neurodevelopmental effects of early infant exposure to psychostimulants via breast milk remain under‐researched [53, 54].

Exclusively breastfed infants were exposed to anxiolytics, hypnotics, and sedatives to a minimal degree. The prevalence of anxiolytic exposure remained stable in the study period, while hypnotics and sedatives increased by about 70%. The metabolites of benzodiazepines may accumulate, and the half‐life in infants is prolonged [55]. For these reasons, the American Academy of Paediatrics discourages chronic use during breastfeeding [56]. Anxiety and related disorders have overlapping symptoms with depression and are often comorbid with depression, and several antidepressants, including SSRIs and serotonin norepinephrine reuptake inhibitors, are often the first line for the treatment of anxiety and related disorders [57, 58].

### 4.5 Potential Factors Associated With Psychotropic Medication Use During Breastfeeding

We found that psychotropic exposure was inversely associated with the duration of breastfeeding. Our findings are consistent with a Norwegian study examining the impact of antidepressant treatment on breastfeeding [31]. The decision to take medications is mainly influenced by maternal underlying disorders, supported by our finding that the largest difference in exposure prevalence was observed in women with a previous psychiatric diagnosis and prior psychotropic medication treatment. Mothers with mental disorders may choose not to initiate breastfeeding or may discontinue breastfeeding if already initiated. Conversely, some mothers may decide to breastfeed but refrain from taking psychotropic medications [31]. Additionally, some antidepressants, antipsychotics, and psychostimulants, like clonidine and guanfacine, may disturb breast milk secretion [59, 60].

## 5 Conclusion

In this large population‐based study, we found that about 17.6 in 1000 exclusively breastfed infants were potentially exposed to psychotropic prescriptions via breastfeeding. The prevalence of psychotropic medication exposure increased during the study period, except for anxiolytics, and the prescription pattern observed suggests that medications were largely prescribed in accordance with changes in clinical guidelines and recommendations. Our findings highlight the need for better evidence on the safety of psychotropic medication during breastfeeding and a clinical focus on the weighing of benefits and harm for both the mother and the child.

## Author Contributions

Drs. Xiaoqin Liu and Mette Bliddal conceived and designed the study, drafted the first draft of the manuscript, and critically reviewed and revised the manuscript. Dr. Kathrine Bang Madsen conceived and designed the study, supervised the data analysis, and critically reviewed and revised the manuscript. Dr. Jin Liang Zhu conducted the data analysis and critically reviewed and revised the manuscript. Drs. Trine Munk‐Olsen, Per Damkier, Angela Lupattelli, Helga Zoega, Hedvig Nordeng, Veerle Bergink, Mette‐Marie Zacher Kjeldsen, Merete Lund Mægbæk, and Malene Galle Madsen critically reviewed and revised the manuscript. All authors approved the final manuscript as submitted and agreed to be accountable for all aspects of the work.

## Ethics Statement

The study was approved by the Danish Data Protection Agency and Statistics Denmark's Scientific Board. Informed consent and ethical approval from the Ethics Committee are not required for studies based on anonymised registry data in Denmark.

## Conflicts of Interest

The authors declare no conflicts of interest.

## Supporting information


**Table S1:** Maternal and child characteristics of infants included and excluded from the study.
**Table S2:** The Anatomical Therapeutic Chemical Classification code for drug classes and the most commonly prescribed individual drug agents.
**Table S3:** The ICD‐10 codes for other mental illnesses up to 5 years before childbirth.
**Table S4:** Counts and prevalence proportion of psychotropic medication use among exclusively breastfeeding women in Denmark, 2012–2022.
**Table S5:** Prevalence (95% CI) of psychotropic medication exposure among 446,573 exclusively breastfed infants in Denmark during 2012–2022, defining psychotropic medication exposure as maternal prescription fills during the 7 days prior to childbirth until the end of exclusive breastfeeding.
**Table S6:** Prevalence (95% CI) of psychotropic medication exposure among 446,573 exclusively breastfed infants in Denmark during 2012–2022, defining psychotropic medication exposure as maternal prescription fills from the date of birth to 14 days before the end of exclusive breastfeeding.
**Figure S1:** Trends in exclusive breastfeeding prevalence in Denmark, 2012–2022.

## Data Availability

The data that support the findings of this study are available from Danish Health Data Authority and Statistics Denmark. Restrictions apply to the availability of these data, which were used under license for this study. Data are available from the author(s) with the permission of Danish Health Data Authority and Statistics Denmark.
