# Supplementary material for: Psychotropic Medication Exposure via Breast Milk: A Population‐Based Descriptive Study in Denmark
Source: Paediatr Perinat Epidemiol. 2025 Sep 11;39(7):612–25. doi: 10.1111/ppe.70074 (PMC12574482; doi:10.1111/ppe.70074)

**Supplementary materials**

**eMethods**

**Definition of birth outcomes**

We defined cesarean delivery based on the ICD-10 code O82 or procedure codes KMCA10-KMCA12 from the Danish Patient Register. We considered a child was born preterm if the child was born with a gestational age < 37 weeks. We defined low birth weight as < 2500 g. small for gestational age (i.e., a birth weight below the 10th percentile of birth weight by gestational age and sex. Information on preterm birth and low birth weight was identified from the Danish Medical Birth Register.

**eTable 1.** Maternal and child characteristics of infants included and excluded from the study.

|  | **Infants included in the study (N=446,573)** | **Infants excluded from the study (N=209,994)** |
| --- | --- | --- |
| **Maternal age at childbirth (years)** |  |  |
| <25 | 48,013 (10.8) | 2,0561 (9.8) |
| 25–29 | 150,409 (33.7) | 65,810 (31.3) |
| 30–34 | 157,705 (35.3) | 76,083 (36.2) |
| ≥35 | 90,446 (20.3) | 47,540 (22.6) |
| **Primiparous** |  |  |
| Yes | 209,440 (46.9) | 88,899 (42.3) |
| No | 237,133 (53.1) | 121,095 (57.7) |
| **Pregnancy** |  |  |
| Singletons | 430,685 (96.4) | 196579 (93.6) |
| Multiple births | 15,888 (3.6) | 13415 (6.4) |
| **Mother's country of origin** |  |  |
| Danish | 359,407 (80.5) | 165,058 (78.6) |
| Non-Danish, including missing | 87,166 (19.5) | 44,936 (21.4) |
| **Maternal smoking during pregnancy** |  |  |
| No | 392,660 (87.9) | 183,039 (87.2) |
| Yes | 40,000 (9.0) | 19,489 (9.3) |
| Missing | 13,913 (3.1) | 7,466 (3.6) |
| **Maternal marital status in the year of delivery** |  |  |
| Married or cohabiting | 411,991 (92.3) | 188,389 (89.7) |
| Single, divorced, or widowed ^a^ | 34,582 (7.7) | 21,605 (10.3) |
| **Maternal education** |  |  |
| Mandatory school, including missing | 69,692 (15.6) | 38,321 (18.2) |
| High school or vocational school | 159,422 (35.7) | 70,436 (33.5) |
| College or university | 217,459 (48.7) | 101,237 (48.2) |
| **Cesarean section** |  |  |
| No | 358,601 (80.3) | 161,038 (76.7) |
| Yes | 87,972 (19.7) | 48,956 (23.3) |
| **Preterm birth** |  |  |
| No | 419,342 (93.9) | 191,312 (91.1) |
| Yes | 23,504 (5.3) | 15,776 (7.5) |
| Missing | 3,727 (0.8) | 2,906 (1.4) |
| **Low birth weight** |  |  |
| No | 423,888 (94.9) | 193,604 (92.2) |
| Yes | 17,863 (4.0) | 12,758 (6.1) |
| Missing | 4,822 (1.1) | 3,632 (1.7) |
| **Maternal psychiatric diagnosis within 5 years before childbirth** |  |  |
| No | 417,268 (93.4) | 195,330 (93.0) |
| Yes | 29,305 (6.6) | 14,664 (7.0) |
| **Substance abuse disorder** |  |  |
| No | 444,066 (99.4) | 208,715 (99.4) |
| Yes | 2,507 (0.6) | 1,279 (0.6) |
| **Schizophrenia** |  |  |
| No | 445,063 (99.7) | 208,999 (99.5) |
| Yes | 1,510 (0.3) | 995 (0.5) |
| **Mood disorders** |  |  |
| No | 439,038 (98.3) | 205,914 (98.1) |
| Yes | 7535 (1.7) | 4080 (1.9) |
| **Anxiety and stress-related disorders** |  |  |
| No | 431,983 (96.7) | 202,892 (96.6) |
| Yes | 14,590 (3.3) | 7,102 (3.4) |
| **Personality disorders** |  |  |
| No | 441,590 (98.9) | 207,509 (98.8) |
| Yes | 4,983 (1.1) | 2,485 (1.2) |
| **Autism spectrum disorders** |  |  |
| No | 446,364 (100.0) | 209,869 (99.9) |
| Yes | 209 (<0.1) | 125 (0.1) |
| **Behavioral and emotional disorders with onset usually occurring in childhood and adolescence** |  |  |
| No | 444,283 (99.5) | 208,821 (99.4) |
| Yes | 2,290 (0.5) | 1,173 (0.6) |
| **Other psychiatric disorders** |  |  |
| No | 441,951 (99.0) | 207,703 (98.9) |
| Yes | 4,622 (1.0) | 2291 (1.1) |
| **Any psychotropic medication within 5 years before childbirth** |  |  |
| No | 380,649 (85.2) | 176,622 (84.1) |
| Yes | 65,924 (14.8) | 33,372 (15.9) |
| **Antipsychotics within 5 years before childbirth** |  |  |
| No | 436,771 (97.8) | 204,414 (97.3) |
| Yes | 9,802 (2.2) | 5,580 (2.7) |
| **Anxiolytics within 5 years before childbirth** |  |  |
| No | 435,114 (97.4) | 203,705 (97.0) |
| Yes | 11,459 (2.6) | 6,289 (3.0) |
| **Hypnotics and sedatives within 5 years before childbirth** |  |  |
| No | 427,168 (95.7) | 200,122 (95.3) |
| Yes | 19,405 (4.3) | 9,872 (4.7) |
| **Antidepressants** **within 5 years before childbirth** |  |  |
| No | 400,832 (89.8) | 186,632 (88.9) |
| Yes | 45,741 (10.2) | 23,362 (11.1) |
| **Psychostimulants within 5 years before childbirth** |  |  |
| No | 441,278 (98.8) | 207,343 (98.7) |
| Yes | 5,295 (1.2) | 2,651 (1.3) |

**eTable 2.** The Anatomical Therapeutic Chemical Classification code for drug classes and the most commonly prescribed individual drug agents

| **Drug classes** | **ATC code** |
| --- | --- |
| **Antipsychotics** | **N05A** |
| *Quetiapine* | N05AH04 |
| *Olanzapine* | N05AH03 |
| *Chlorprothixene* | N05AF03 |
| *Risperidone* | N05AX08 |
| *Lithium* | N05AN01 |
| **Anxiolytics** | **N05B** |
| *Oxazepam* | N05BA04 |
| *Diazepam* | N05BA01 |
| *Alprazolam* | N05BA12 |
| *Clobazam* | N05BA09 |
| **Hypnotics and sedatives** | **N05C** |
| *Zopiclone* | N05CF01 |
| *Zolpidem* | N05CF02 |
| *Melatonin* | N05CH01 |
| *Triazolam* | N05CD05 |
| **Antidepressants** | **N06A** |
| *Sertraline* | N06AB06 |
| *Citalopram* | N06AB04 |
| *Venlafaxine* | N06AX16 |
| *Escitalopram* | N06AB10 |
| *Nortriptyline* | N06AA10 |
| **Psychostimulants** | **N06B** |
| *Methylphenidate* | N06BA04 |
| *Lisdexamfetamine* | N06BA12 |
| *Atomoxetine* | N06BA09 |

Abbreviation: ATC code, Anatomical Therapeutic Chemical Classification code.

**eTable 3.** The ICD-10 codes for other mental illnesses up to five years before childbirth

| **Name of disorders** | **ICD-10 codes** |
| --- | --- |
| Any psychiatric disorders | F00–F99 |
| Substance abuse disorder | F10–F19 |
| Schizophrenia and related disorders, abbreviated hereafter as psychotic disorders | F20–F29 |
| Mood disorders | F30–39 |
| Anxiety and stress-related disorders | F40–F48 |
| Personality disorders | F60–F69 |
| Autism spectrum disorders | F84 |
| Behavioural and emotional disorders with onset usually occurring in childhood and adolescence | F90–F98 |
| Others | F00–F09, F50–F59, F70–F79, F80–F83, F88–F89, F99 |

Abbreviation: ICD-10, International Statistical Classification of Diseases and Related Health Problems,10th Revision

**eTable 4.** Counts and prevalence proportion of psychotropic medication use among exclusively breastfeeding women in Denmark, 2012–2022.

| **Characteristics** | **No. of infants** | **Any psychotropic medications** | | **Antipsychotics** | | **Anxiolytics** | | **Hypnotics and sedatives** | | **Antidepressants** | | **Psychostimulants** | |
| --- | --- | --- | --- | --- | --- | --- | --- | --- | --- | --- | --- | --- | --- |
|  |  | **N** | **/1,000 infants** | **N** | **/1,000 infants** | **N** | **/1,000 infants** | **N** | **/1,000 infants** | **N** | **/1,000 infants** | **N** | **/1,000 infants** |
| **Overall** | 446,573 | 7,882 | 17.6 | 472 | 1.1 | 309 | 0.7 | 577 | 1.3 | 6,685 | 15.0 | 457 | 1.0 |
| **Duration of breastfeeding (months)** |  |  |  |  |  |  |  |  |  |  |  |  |  |
| <1 | 87,745 | 260 | 3.0 | 28 | 0.3 | 15 | 0.2 | 34 | 0.4 | 188 | 2.1 | 24 | 0.3 |
| 1-3 | 88,129 | 1,565 | 17.8 | 102 | 1.2 | 63 | 0.7 | 142 | 1.6 | 1,261 | 14.3 | 131 | 1.5 |
| 4-5 | 181,778 | 4,005 | 22.0 | 225 | 1.2 | 146 | 0.8 | 260 | 1.4 | 3,472 | 19.1 | 204 | 1.1 |
| ≥6 | 88,921 | 2,052 | 23.1 | 117 | 1.3 | 85 | 1.0 | 141 | 1.6 | 1,764 | 19.8 | 98 | 1.1 |
| **Maternal age at childbirth (years)** |  |  |  |  |  |  |  |  |  |  |  |  |  |
| <25 | 48,013 | 625 | 13.0 | 55 | 1.1 | 23 | 0.5 | 29 | 0.6 | 485 | 10.1 | 89 | 1.9 |
| 25–29 | 150,409 | 2,245 | 14.9 | 127 | 0.8 | 79 | 0.5 | 146 | 1.0 | 1,882 | 12.5 | 168 | 1.1 |
| 30–34 | 157,705 | 2,952 | 18.7 | 170 | 1.1 | 116 | 0.7 | 230 | 1.5 | 2,528 | 16.0 | 136 | 0.9 |
| ≥35 | 90,446 | 2,060 | 22.8 | 120 | 1.3 | 91 | 1.0 | 172 | 1.9 | 1,790 | 19.8 | 64 | 0.7 |
| **Primiparous** |  |  |  |  |  |  |  |  |  |  |  |  |  |
| Yes | 209,440 | 3,663 | 17.5 | 220 | 1.1 | 137 | 0.7 | 312 | 1.5 | 3,021 | 14.4 | 259 | 1.2 |
| No | 237,133 | 4,219 | 17.8 | 252 | 1.1 | 172 | 0.7 | 265 | 1.1 | 3,664 | 15.5 | 198 | 0.8 |
| **Pregnancy** |  |  |  |  |  |  |  |  |  |  |  |  |  |
| Singletons | 430,685 | 7,676 | 17.8 | 464 | 1.1 | 297 | 0.7 | 553 | 1.3 | 6,518 | 15.1 | 451 | 1.0 |
| Multiple births | 15,888 | 206 | 13.0 | 8 | 0.5 | 12 | 0.8 | 24 | 1.5 | 167 | 10.5 | 6 | 0.4 |
| **Mother's country of origin** |  |  |  |  |  |  |  |  |  |  |  |  |  |
| Danish | 359,407 | 6,837 | 19.0 | 378 | 1.1 | 257 | 0.7 | 469 | 1.3 | 5,815 | 16.2 | 420 | 1.2 |
| Non-Danish, including missing | 87,166 | 1,045 | 12.0 | 94 | 1.1 | 52 | 0.6 | 108 | 1.2 | 870 | 10.0 | 37 | 0.4 |
| **Maternal smoking during pregnancy** |  |  |  |  |  |  |  |  |  |  |  |  |  |
| No | 392,660 | 6,589 | 16.8 | 380 | 1.0 | 264 | 0.7 | 520 | 1.3 | 5,637 | 14.4 | 320 | 0.8 |
| Yes | 40,000 | 1,042 | 26.1 | 78 | 2.0 | 36 | 0.9 | 38 | 1.0 | 834 | 20.9 | 122 | 3.1 |
| Missing | 13,913 | 251 | 18.0 | 14 | 1.0 | 9 | 0.6 | 19 | 1.4 | 214 | 15.4 | 15 | 1.1 |
| **Maternal marital status in the year of delivery** |  |  |  |  |  |  |  |  |  |  |  |  |  |
| Married or cohabiting | 411,991 | 7,074 | 17.2 | 403 | 1.0 | 277 | 0.7 | 531 | 1.3 | 6063 | 14.7 | 360 | 0.9 |
| Single, divorced, or widowed ^a^ | 34,582 | 808 | 23.4 | 69 | 2.0 | 32 | 0.9 | 46 | 1.3 | 622 | 18.0 | 97 | 2.8 |
| **Maternal education** |  |  |  |  |  |  |  |  |  |  |  |  |  |
| Mandatory school, including missing | 69,692 | 1,385 | 19.9 | 131 | 1.9 | 47 | 0.7 | 63 | 0.9 | 1,079 | 15.5 | 170 | 2.4 |
| High school or vocational school | 159,422 | 2,796 | 17.5 | 163 | 1.0 | 107 | 0.7 | 142 | 0.9 | 2,403 | 15.1 | 180 | 1.1 |
| College or university | 217,459 | 3,701 | 17.0 | 178 | 0.8 | 155 | 0.7 | 372 | 1.7 | 3,203 | 14.7 | 107 | 0.5 |
| **Caesarean section** |  |  |  |  |  |  |  |  |  |  |  |  |  |
| No | 358,601 | 6,125 | 17.1 | 358 | 1.0 | 232 | 0.6 | 458 | 1.3 | 5,204 | 14.5 | 358 | 1.0 |
| Yes | 87,972 | 1,757 | 20.0 | 114 | 1.3 | 77 | 0.9 | 119 | 1.4 | 1,481 | 16.8 | 99 | 1.1 |
| **Preterm birth** |  |  |  |  |  |  |  |  |  |  |  |  |  |
| No | 419,342 | 7,117 | 17.0 | N/A | N/A | N/A | N/A | N/A | N/A | 6,007 | 14.3 | N/A | N/A |
| Yes | 23,504 | 728 | 31.0 | N/A | N/A | N/A | N/A | N/A | N/A | 648 | 27.6 | N/A | N/A |
| Missing | 3,727 | 37 | 9.9 | N/A | N/A | N/A | N/A | N/A | N/A | 30 | 8.0 | N/A | N/A |
| **Low birth weight** |  |  |  |  |  |  |  |  |  |  |  |  |  |
| No | 423,888 | 7,357 | 17.4 | N/A | N/A | N/A | N/A | 540 | 1.3 | 6,241 | 14.7 | N/A | N/A |
| Yes | 17,863 | 480 | 26.9 | N/A | N/A | N/A | N/A | 32 | 1.8 | 408 | 22.8 | N/A | N/A |
| Missing | 4,822 | 45 | 9.3 | N/A | N/A | N/A | N/A | 5 | 1.0 | 36 | 7.5 | N/A | N/A |
| **Maternal psychiatric diagnosis within 5 years before childbirth** |  |  |  |  |  |  |  |  |  |  |  |  |  |
| No | 417,268 | 4,656 | 11.2 | 152 | 0.4 | 225 | 0.5 | 465 | 1.1 | 3921 | 9.4 | 230 | 0.6 |
| Yes | 29,305 | 3,226 | 110.1 | 320 | 10.9 | 84 | 2.9 | 112 | 3.8 | 2764 | 94.3 | 227 | 7.7 |
| **Substance abuse disorder** |  |  |  |  |  |  |  |  |  |  |  |  |  |
| No | 444,066 | 7,789 | 17.5 | 456 | 1.0 | N/A | N/A | N/A | N/A | 6,616 | 14.9 | 447 | 1.0 |
| Yes | 2,507 | 93 | 37.1 | 16 | 6.4 | N/A | N/A | N/A | N/A | 69 | 27.5 | 10 | 4.0 |
| **Schizophrenia** |  |  |  |  |  |  |  |  |  |  |  |  |  |
| No | 445,063 | 7,711 | 17.3 | 389 | 0.9 | 303 | 0.7 | 565 | 1.3 | 6,596 | 14.8 | 447 | 1.0 |
| Yes | 1,510 | 171 | 113.2 | 83 | 55.0 | 6 | 4.0 | 12 | 7.9 | 89 | 58.9 | 10 | 6.6 |
| **Mood disorders** |  |  |  |  |  |  |  |  |  |  |  |  |  |
| No | 439,038 | 6,416 | 14.6 | 319 | 0.7 | 275 | 0.6 | 532 | 1.2 | 5,355 | 12.2 | 423 | 1.0 |
| Yes | 7,535 | 1,466 | 194.6 | 153 | 20.3 | 34 | 4.5 | 45 | 6.0 | 1,330 | 176.5 | 34 | 4.5 |
| **Anxiety and stress-related disorders** |  |  |  |  |  |  |  |  |  |  |  |  |  |
| No | 431,983 | 6,329 | 14.7 | 350 | 0.8 | 254 | 0.6 | 519 | 1.2 | 5,299 | 12.3 | 400 | 0.9 |
| Yes | 14,590 | 1,553 | 106.4 | 122 | 8.4 | 55 | 3.8 | 58 | 4.0 | 1,386 | 95.0 | 57 | 3.9 |
| **Personality disorders** |  |  |  |  |  |  |  |  |  |  |  |  |  |
| No | 441,590 | 7,394 | 16.7 | 405 | 0.9 | 301 | 0.7 | 566 | 1.3 | 6,274 | 14.2 | 424 | 1.0 |
| Yes | 4,983 | 488 | 97.9 | 67 | 13.4 | 8 | 1.6 | 11 | 2.2 | 411 | 82.5 | 33 | 6.6 |
| **Autism spectrum disorders** |  |  |  |  |  |  |  |  |  |  |  |  |  |
| No | 446,364 | 7,858 | 17.6 | N/A | N/A | N/A | N/A | N/A | N/A | 6,665 | 14.9 | N/A | N/A |
| Yes | 209 | 24 | 114.8 | N/A | N/A | N/A | N/A | N/A | N/A | 20 | 95.7 | N/A | N/A |
| **Behavioral and emotional disorders with onset usually occurring in childhood and adolescence** |  |  |  |  |  |  |  |  |  |  |  |  |  |
| No | 444,283 | 7,626 | 17.2 | 452 | 1.0 | N/A | N/A | 569 | 1.3 | 6,585 | 14.8 | 297 | 0.7 |
| Yes | 2,290 | 256 | 111.8 | 20 | 8.7 | N/A | N/A | 8 | 3.5 | 100 | 43.7 | 160 | 69.9 |
| **Other psychiatric disorders** |  |  |  |  |  |  |  |  |  |  |  |  |  |
| No | 441,951 | 7,516 | 17.0 | 429 | 1.0 | 295 | 0.7 | 552 | 1.2 | 6,379 | 14.4 | 431 | 1.0 |
| Yes | 4,622 | 366 | 79.2 | 43 | 9.3 | 14 | 3.0 | 25 | 5.4 | 306 | 66.2 | 26 | 5.6 |
| **Any psychotropic medication within 5 years before childbirth** |  |  |  |  |  |  |  |  |  |  |  |  |  |
| No | 380,649 | 864 | 2.3 | 75 | 0.2 | 121 | 0.3 | 289 | 0.8 | 525 | 1.4 | 11 | 0.0 |
| Yes | 65,924 | 7,018 | 106.5 | 397 | 6.0 | 188 | 2.9 | 288 | 4.4 | 6160 | 93.4 | 446 | 6.8 |
| **Antipsychotics within 5 years before childbirth** |  |  |  |  |  |  |  |  |  |  |  |  |  |
| No | 436,771 | 6,453 | 14.8 | 137 | 0.3 | 266 | 0.6 | 508 | 1.2 | 5,583 | 12.8 | 363 | 0.8 |
| Yes | 9,802 | 1,429 | 145.8 | 335 | 34.2 | 43 | 4.4 | 69 | 7.0 | 1,102 | 112.4 | 94 | 9.6 |
| **Anxiolytics within 5 years before childbirth** |  |  |  |  |  |  |  |  |  |  |  |  |  |
| No | 435,114 | 6,640 | 15.3 | 367 | 0.8 | 192 | 0.4 | 514 | 1.2 | 5,641 | 13.0 | 399 | 0.9 |
| Yes | 11,459 | 1,242 | 108.4 | 105 | 9.2 | 117 | 10.2 | 63 | 5.5 | 1,044 | 91.1 | 58 | 5.1 |
| **Hypnotics and sedatives within 5 years before childbirth** |  |  |  |  |  |  |  |  |  |  |  |  |  |
| No | 427,168 | 6,244 | 14.6 | 318 | 0.7 | 245 | 0.6 | 389 | 0.9 | 5,400 | 12.6 | 307 | 0.7 |
| Yes | 19,405 | 1,638 | 84.4 | 154 | 7.9 | 64 | 3.3 | 188 | 9.7 | 1,285 | 66.2 | 150 | 7.7 |
| **Antidepressants** **within 5 years before childbirth** |  |  |  |  |  |  |  |  |  |  |  |  |  |
| No | 400,832 | 1,415 | 3.5 | 195 | 0.5 | 171 | 0.4 | 395 | 1.0 | 605 | 1.5 | 263 | 0.7 |
| Yes | 45,741 | 6,467 | 141.4 | 277 | 6.1 | 138 | 3.0 | 182 | 4.0 | 6,080 | 132.9 | 194 | 4.2 |
| **Psychostimulants within 5 years before childbirth** |  |  |  |  |  |  |  |  |  |  |  |  |  |
| No | 441,278 | 7,162 | 16.2 | 442 | 1.0 | 300 | 0.7 | 557 | 1.3 | 6,375 | 14.4 | 18 | 0.0 |
| Yes | 5,295 | 720 | 136.0 | 30 | 5.7 | 9 | 1.7 | 20 | 3.8 | 310 | 58.5 | 439 | 82.9 |

N/A: not applicable due to less than 5 cases or not relevant.

a including missing

**eTable 5.** Prevalence (95%CI) of psychotropic medication exposure among 446,573 exclusively breastfed infants in Denmark during 2012–2022, defining psychotropic medication exposure as maternal prescription fills during the 7 days prior to childbirth until the end of exclusive breastfeeding

| **Year** | **N** | **Number of exposed infants** | **Prevalence (95%CI), per 1,000 infants** |
| --- | --- | --- | --- |
| 2012 – 2022 | 446,573 | 9,003 | 20.2 (19.7–20.6) |
| 2012 | 32,616 | 580 | 17.8 (16.4–19.2) |
| 2013 | 34,656 | 609 | 17.6 (16.2–19.1) |
| 2014 | 40,452 | 738 | 18.2 (17.0–19.6) |
| 2015 | 43,208 | 764 | 17.7 (16.4–19.0) |
| 2016 | 44,731 | 871 | 19.5 (18.1–20.9) |
| 2017 | 44,726 | 826 | 18.5 (17.3–19.8) |
| 2018 | 42,637 | 854 | 20.0 (18.7–21.4) |
| 2019 | 42,580 | 846 | 19.9 (18.5–21.3) |
| 2020 | 40,380 | 931 | 23.1 (21.6–24.6) |
| 2021 | 44,064 | 1,062 | 24.1 (22.7–25.5) |
| 2022 | 36,523 | 922 | 25.2 (23.7–26.9) |

**eTable 6.** Prevalence (95%CI) of psychotropic medication exposure among 446,573 exclusively breastfed infants in Denmark during 2012–2022, defining psychotropic medication exposure as maternal prescription fills from the date of birth to 14 days before the end of exclusive breastfeeding

| **Year** | **N** | **Number of exposed infants** | **Prevalence (95%CI), per 1,000 infants** |
| --- | --- | --- | --- |
| 2012 – 2022 | 446,573 | 7,527 | 15.9 (14.7, 17.1) |
| 2012 | 32,616 | 473 | 14.5 (13.2, 15.9) |
| 2013 | 34,656 | 503 | 14.5 (13.3, 15.8) |
| 2014 | 40,452 | 613 | 15.2 (14.0,16.4) |
| 2015 | 43,208 | 627 | 14.5 (13.4, 15.7) |
| 2016 | 44,731 | 719 | 16.1 (14.9, 17.3) |
| 2017 | 44,726 | 666 | 14.9 (13.8, 16.1) |
| 2018 | 42,637 | 726 | 17.0 (15.8,18.3) |
| 2019 | 42,580 | 706 | 16.6 (15.4, 17.8) |
| 2020 | 40,380 | 756 | 18.7 (17.4, 20.1) |
| 2021 | 44,064 | 875 | 19.9 (18.6, 21.2) |
| 2022 | 36,523 | 863 | 23.6 (22.1, 25.2) |

**eFigure.** Trends in exclusive breastfeeding prevalence in Denmark, 2012–2022


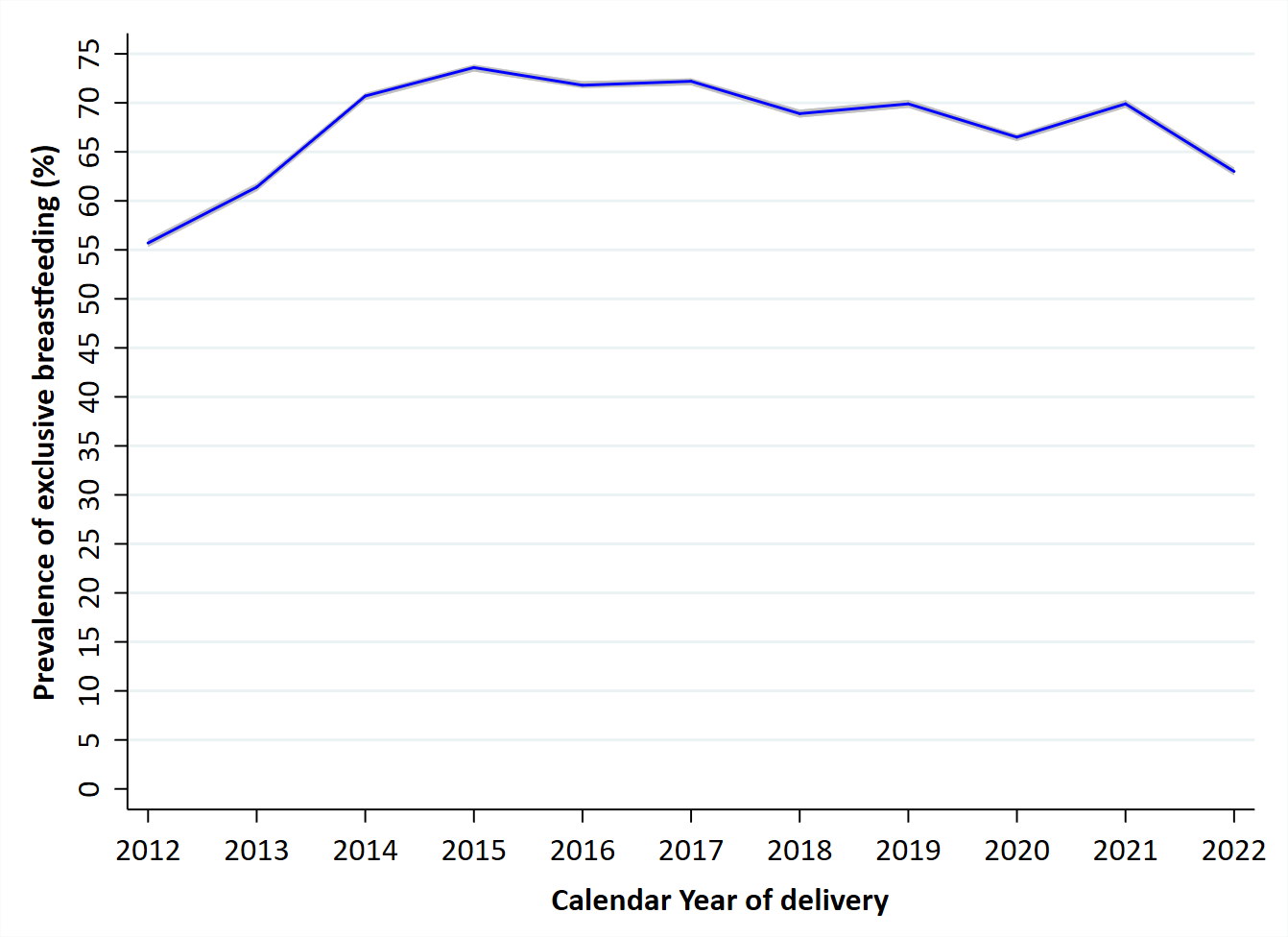

Supplement: Supplementary file 1 — Table S1: Maternal and child characteristics of infants included and excluded from the study. Table S2: The Anatomical Therapeutic Chemical Classification code for drug classes and the most commonly prescribed individual drug agents. Table S3: The ICD‐10 codes for other mental illnesses up to 5 years before childbirth. Table S4: Counts and prevalence proportion of psychotropic medication use among exclusively breastfeeding women in Denmark, 2012–2022. Table S5: Prevalence (95% CI) of psychotropic medication exposure among 446,573 exclusively breastfed infants in Denmark during 2012–2022, defining psychotropic medication exposure as maternal prescription fills during the 7 days prior to childbirth until the end of exclusive breastfeeding. Table S6: Prevalence (95% CI) of psychotropic medication exposure among 446,573 exclusively breastfed infants in Denmark during 2012–2022, defining psychotropic medication exposure as maternal prescription fills from the date of birth to 14 days before the end of exclusive breastfeeding. Figure S1: Trends in exclusive breastfeeding prevalence in Denmark, 2012–2022. [file PPE-39-612-s001.docx]
